# An evaluation of digital intervention for perinatal depression and anxiety: A systematic review

**DOI:** 10.3934/publichealth.2024025

**Published:** 2024-04-15

**Authors:** Siti Roshaidai Mohd Arifin, Amalia Kamaruddin, Noor Azimah Muhammad, Mohd Said Nurumal, Hazwani Mohd Mohadis, Nik Hazlina Nik Hussain, Shanti Wardaningsih

**Affiliations:** 1 Kulliyyah of Nursing, International Islamic University Malaysia; 2 Faculty of Medicine, Universiti Kebangsaan Malaysia; 3 Kulliyyah of Information and Communication Technology, International Islamic University Malaysia; 4 School of Medical Sciences, Universiti Sains Malaysia; 5 Universitas Muhammadiyah Yogyakarta, Kampus Terpadu UMY, JI. Brawijaya, Kasihan, Bantul, Yogyakarta, Indonesia

**Keywords:** depression, anxiety, perinatal, partner, intervention, digital

## Abstract

Digital intervention has been shown to be helpful in improving perinatal mental health. However, the design characteristics of such interventions have not been systematically reviewed. Considering that a lack of support—especially from a partner—is one of the major contributing factors to perinatal depression and anxiety, it is crucial to determine whether digital interventions have included partner participation. In this review, we systematically examined the design characteristics of digital interventions related to perinatal depression and anxiety and aimed to determine whether partner participation was incorporated as part of the interventions. Based on the PRISMA 2020 guidelines, five databases (PubMed, EBSCO, Cochrane, ProQuest, and Scopus) were searched. Narrative results of design characteristics were developed to provide a framework for the design and evaluation of the studies. A total of 12 intervention studies from China, Sweden, Australia, New Zealand, Singapore, Norway, and the United Kingdom were included. Across all studies, internet cognitive behavioral therapy and mindfulness therapy were overwhelmingly utilized as the major intervention approaches. While all studies reported reduced depressive symptoms after the intervention, only four studies reported subsequent decreased levels of both depressive and anxiety symptoms. Only one study included partner support in the intervention. Cognitive behavioral therapy and mindfulness therapy, two of the most common intervention approaches, were found to be effective in alleviating perinatal depression and anxiety. Partner participation should be prioritized in designing digital interventions to ensure comprehensive and easily accessible social support for persons in need.

## 1. Introduction

Perinatal depression and anxiety affect 20–24% of pregnant and postnatal persons globally [1]–[4]. Apart from affecting individual function, perinatal depression, and anxiety can also influence birth outcomes, breastfeeding practice, and birthing person-infant interactions, all of which affect a child's growth and development [5]–[7]. In many instances, pregnant and postnatal persons are reluctant to discuss their mental health with healthcare practitioners (HCPs) due to a fear of being judged, labeled, or stigmatized [8].

Various factors contribute to the development of perinatal depression and anxiety, including a family or personal history of anxiety or depression, pregnancy complications due to medical comorbidities (e.g., gestational diabetes mellitus or hypertension in pregnancy), and financial difficulties. However, the major contributing factor has been identified as a lack of partner support [9]. Receiving support from a partner (such as voluntarily helping with the housework, being a source of financial support [instrumental support], providing emotional support, and responding positively during challenging times, such as during the Covid-19 pandemic) are of benefit to pregnant persons in terms of their mental well-being and lessen the risk of perinatal depression and anxiety [10],[11].

To date, existing partner-inclusive interventions for perinatal depression and anxiety primarily comprise face-to-face group classes delivered by HCPs. Among the approaches that can be considered is a digital health intervention [12]. Certain barriers to treating perinatal depression and anxiety have been identified, including stigma, time scheduling, and a lack of information about access to treatment [13]. To address these obstacles, digital approaches offer an alternative to conventional method [14],[15]. Digital approaches have been used to deliver interventions aimed at alleviating perinatal depression and anxiety, especially in Western countries [16]–[18].

More specifically, several digital applications targeting perinatal mental health—namely, an eMBI (the Mindmom application) and Mamma Mia—have demonstrated modest success in reducing symptoms of depression and anxiety [16]–[19]. A recent study has shown that digital intervention is beneficial and complementary to screening, prevention, and follow-up programs for persons with perinatal mental health issues, especially those reluctant to seek professional help [18]. Moreover, digital interventions confer the choice of anonymity, enabling the persons to overcome stigma and obtain help from HCPs. Digital interventions have also been recommended as solutions to overcome geographical, financial, and psychological limitations [20].

To improve the sustainability of and adherence to intervention courses, it is recommended that the intervention content be devised or adapted to promote a person empowerment (self-care management). It should be carefully tailored to the person's self-care needs or preferences, including the need for support from their partner. Zingg A et al. [17] conducted two focus group sessions with nine perinatal persons, as well as 10 semi-structured interviews with patients obtaining care in maternal-fetal medicine clinics, to understand their views on the use of digital technology in meeting mental health information needs. The findings highlighted that digital interventions should focus on needs that are specific and relevant to perinatal mental health, as well as offer personalization and coping strategies to ensure the effective self-care management of mental health.

Despite the extensive number of existing digital interventions, neither their content nor their functions have been systematically reviewed. In addition, because a lack of partner support can lead to perinatal depression and anxiety, it is important to determine whether the existing digital interventions include partner participation. Partner support in this review refers to both married and unmarried couples. Therefore, we aimed to, first, analyze the design characteristics (including the content and functions/approaches) of existing digital interventions related to mental health issues, particularly depression and anxiety in perinatal persons, and second, determine whether the element of spousal support was included in the interventions.

## 2. Materials and methods

### 2.1. Inclusion and exclusion criteria

This review was conducted and reported based on the Preferred Reporting Items for Systematic Reviews and Meta-Analyses (PRISMA) guidelines [21]. We aimed to examine the effectiveness of partner-inclusive interventions in preventing perinatal depression and anxiety in perinatal persons. The following specific keywords were used: (“maternal mental health” OR depression OR anxiety OR emotion* OR disorders OR distress) AND (antenatal OR prenatal OR perinatal OR postnatal OR postpartum) AND (program OR intervention) AND (electronic OR online OR computer OR web OR website OR mobile OR internet). Five relevant databases—PubMed, EBSCO Discovery Service (EDS), Cochrane Library, ProQuest Health & Medical Complete, and Scopus—were used in a search that covered the period 2016–2023.

**Figure 1.**
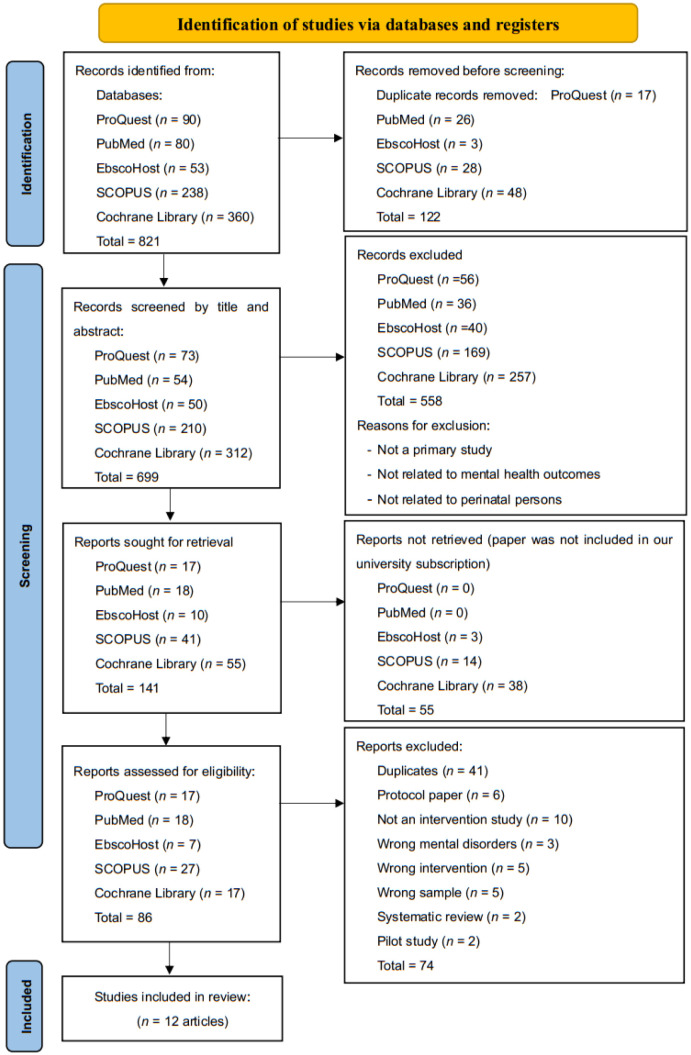
Preferred Reporting Items for Systematic Reviews and Meta-Analyses (PRISMA).

The eight inclusion criteria for the studies were as follows: (i) The subjects were perinatal persons (from pregnancy to one year after childbirth); (ii) the intervention was designed to prevent or improve mental health issues, particularly depression and/or anxiety; (iii) mental health outcomes were reported; (iv) only primary studies were included; (v) pre-and post-study results were available and/or a randomized controlled trial study was conducted; (vi) the study was published in English or Malay; (vii) the study was published between 2016 and 2023; and (viii) the study involved only digital intervention. The study period from 2016 to 2023 was chosen as a continuation of an earlier systematic literature review conducted by Ashford L et al. [15] on the effects of computer- or web-based interventions on perinatal mental health. The six exclusion criteria were as follows: (i) Studies on online consultation; (ii) qualitative studies; (iii) case studies; (iv) vulnerable groups such as immigrants or those suffering intimate partner violence; (v) narrative reviews, systematic reviews, or meta-analyses; and (vi) study protocols. As we aim to report the findings of primary literature, review papers were excluded, as they are typically considered secondary studies. Figure 1 provides an overview of the overall research methodology using PRISMA.

### 2.2. Critical appraisal

To ensure the quality of the included studies, two independent researchers appraised the papers, following the standard quality assessment criteria for evaluating primary research papers [22]. The assessment consisted of a four-point scoring scale (2 = “yes”, 1 = “partial”, 0 = “no”, and “N/A”) for a total of 14 criteria. A higher summary score represents a paper of higher methodological quality.

The following total scores were obtained:

- Total sum = (number of “yes” choices * 2) + (number of “partials” * 1)
- Total possible sum = 28 – (number of “N/A” * 2)
- Summary score = Total sum/Total possible sum

The total score for the quality analysis was 0.93, with a higher score indicating a paper of higher quality (maximum score = 1.00). The scores were hermeneutically derived based on the reviewers' discretion. Based on the results, four of the 12 papers that scored between 0.90 and 1.00 were categorized as high-quality intervention papers. Another seven papers scored above 0.80, indicating good quality, while the remaining paper scored between 0.60 and 0.79 and was categorized as being of average quality.

Most papers obtained lower scores for Criteria 3 (methodology), 5 (randomization methodology), 6 (blinding of the investigator), 7 (blinding of the subject), 11 (estimates of variance for results), and 12 (controlled for confounding). Due to the nature of digital interventions, most studies were unable to fulfill the blinding criteria for investigators and respondents. However, all the studies clearly stated their aims, research design, subject selection, outcomes, appropriate sample sizes, and analysis, as well as reported sufficient results and conclusions supported by tabulated data. As reported by Kmet LM et al. [22], the quality assessment of the included studies was based on the 14 criteria listed in Table 1 below.

## 3. Results

A total of 12 intervention studies were included in this systematic literature review. These papers were screened from 5 databases: PubMed, ProQuest, EDS, Scopus, and Cochrane. The studies came from nine countries, including China (*n* = 4), Sweden (*n* = 2), and Australia (*n* = 2), with one each from New Zealand, Singapore, Norway, and the United Kingdom. The 12 papers were randomized control trials (RCTs), with sample sizes ranging between 27 and 1342. Table 2 presents a summary of all the included studies.

**Table 1.** Quality assessment of included studies.

**Author**	**Aims**	**Design**	**Method**	**Subject**	**Random**	**Blind investigator**	**Blind subject**	**Outcome**	**Sample**	**Analysis**	**Variance**	**Confounding**	**Results**	**Conclusion**	**Overall**
**Sjomark J et al. [23]**	2	2	2	2	2	0	0	2	2	2	2	2	2	2	0.86
**Bear KA et al. [24]**	2	2	2	2	2	0	0	2	2	2	2	2	2	2	0.86
**Sun Y et al. [25]**	2	2	2	2	1	2	0	2	2	2	2	2	2	2	0.89
**Chan KL et al. [26]**	2	2	2	2	2	2	0	2	2	2	2	2	2	2	0.93
**Shorey S et al. [27]**	2	2	2	2	2	2	0	2	2	2	2	2	2	2	0.93
**Haga SM et al. [28]**	2	2	1	2	2	2	0	2	2	2	2	2	2	2	0.89
**Yang M et al. [29]**	2	2	2	2	2	2	0	2	2	2	2	2	2	2	0.93
**Guo L et al. [30]**	2	2	1	2	2	0	0	2	2	2	1	2	2	1	0.75
**Loughnan SA et al. [31]**	2	2	2	2	2	2	0	2	2	2	1	2	2	2	0.89
**Krusche A et al [32]**	2	2	2	2	2	0	0	2	2	2	1	2	2	2	0.82
**Forsell E et al. [33]**	2	2	2	2	2	2	0	2	1	2	2	1	2	2	0.86
**Milgrom J et al. [34]**	2	2	2	2	2	0	2	2	2	2	2	2	2	2	0.93

**Table 2.** Criteria for included studies.

**No.**	**Author**	**Intervention name & format**	**Origin**	**Therapeutic approach**	**Content**	**Study design**	**Sample size**	**Measures/ instruments**	**Assessment period**	**Outcomes**	**Partner elements included?**
**1**	Sjomark J et al. [23]	Internet-based intervention	Sweden	iCBT (web-based)	**Part 1:** Information, psychoeducation, and breathing retraining Vignettes, information on common symptoms, fear, and avoidance Depressive symptoms, significance of relations, and information about reflective listening Exposure, talking about childbirth with others Managing anxiety and depressive symptoms, information about psychological health, life values, and recovery Summary, repetition, and relapse prevention **Part 2:** Introduction to treatment Part 2, psychoeducation regarding PTSD Identify & recognize symptoms, breathing retraining (continued through treatment) In vivo exposure (continued through treatment) Refined in vivo exposure + intro to expressive writing Expressive writing, imaginal exposure Refined imaginal exposure Finding hot spots, recovery Summary, maintaining progress, relapse prevention	Randomized controlled trial (RCT)	Healthy postnatal person: iCBT: *n* = 132 TAU: *n* = 134 *N* = 266	Edinburgh Postnatal Depression Scale (EPDS) Traumatic Event Scale (TES) Satisfaction with Life Scale (SWLS) Ways of Coping Questionnaire (WCQ)	T0: At baseline T1: 8–16 weeks postnatal T2: 6 weeks after randomization T3: 14 weeks after randomization T4: 1 year after randomization	There was a significant main effect of time and quadratic time on the EPDS scale.	No
**2**	Bear KA et al. [24]	Mindfulness app	New Zealand	Smiling Mind app (mobile-based)	10 modules: The Breath Sound and Taste Thoughts Emotions Everyday Mindfulness Curiosity and Beginner's Mind Stress Sleep and Gratitude Relationships Mindful Listening	RCT	Healthy postnatal person: Intervention: *n* = 49 Control: *n* = 50 *N* = 99	DASS-21 Mindful Attention Awareness Scale (MAAS)	T1: Baseline T2: Post-intervention T3: 4 weeks follow-up scores	The results show that the delivery of mindfulness via smartphones could be beneficial not only in reducing postnatal depression, anxiety, and stress but also in enhancing mindful attention and awareness.	No
**3**	Sun Y et al. [25]	Smartphone-based mindfulness	China	Spirit Healing (mobile-based)	Consists of eight sessions: **Week 1:** Understand mindfulness. **Week 2:** Be in the present. **Week 3:** Be mindful of negative emotions. **Week 4:** Accept difficulties. **Week 5:** Thoughts are just thoughts. **Week 6:** Enjoy daily happiness. **Week 7:** Mindful pregnancy and childbirth. **Week 8:** Continued mindfulness practice.	2 parallel-arm RCT	Antenatal person with depression: Intervention: *n* = 84 Control: *n* = 84 *N* = 168	Edinburgh Postnatal Depression Scale (EPDS) Patient Health Questionnaire-9 (PHQ-9)	T1: At baseline T2: 4 weeks after allocation (intermediate period of intervention) T3: 8 weeks after allocation (endpoint of the intervention) T4: 18 weeks after allocation (before childbirth) T5: 6 weeks after delivery	Participants in mindfulness training showed significant reduction in depressive symptoms compared to participants in attention group.	No
**4**	Chan KL et al. [26]	Smartphone-based psycho-education	China	Psychoeducation mobile app (mobile-based)	iParent apps (psychoeducation on): Antenatal care Postnatal care Infant care Child protection	RCT	Healthy antenatal person: Intervention: *n* = 330 Control: *n* = 330 *N* = 660	Edinburgh Postnatal Depression Scale (EPDS)	T1: At baseline T2: Follow-up (post-intervention)	The combination of smartphone-based intervention plus TAU services was effective in reducing postnatal depression at 4 weeks postnatal compared to control condition of TAU only.	No
**5**	Shorey S et al. [27]	Technology-based peer-support	Singapore	Peer support intervention program (PIP) (mobile-based)	Intervention groups' mothers received a technology-based peer-support program for 4 weeks after delivery. In the PIP intervention, correspondence with trained peer volunteer was conducted at least once a week through phone calls, emails, or WhatsApp based on the preference and convenience of each mother. Both sides (peer and mothers) shared experiences of emotional distress during the early postnatal period in the introductory phone session.	Single-blinded 2-group pre-test and post-test RCT	Postnatal person with depression: Intervention: *n* = 69 Control: *n* = 69 *N* = 138	EPDS Patient Health Questionnaire (PHQ-9) State-Trait Anxiety Inventory (STAI) Perceived Social Support for Parenting (PSSP) University of California, Los Angeles Loneliness Scale (ULS)	T1: At birth T1: 1 month postnatal T2: 3 months postnatal	The technology-based PIP was found to be effective in reducing the risk of PND among new mothers and showed a generally positive trend in reducing postnatal anxiety (PNA) and loneliness and in increasing perceived social support.	No
**6**	Haga SM et al. [28]	Internet-based intervention (Mamma Mia)	Norway	Mamma Mia (web-based)	First phase: 11 sessions beginning in the second trimester in gestational weeks (gw) 21–25 and ending in gw 37. Second phase: Starts when the infant is 2–3 weeks old and lasts for 6 weeks (3 sessions/week). Final phase: 10 sessions over an 18-week period. The intervention was delivered by email and interactive websites, combining text, pictures, pre-recorded audio files, and user input. The users must complete all items in the current session, and it takes 10 minutes before they can gain access to the next session.	RCT	Healthy antenatal person: Intervention: *n* = 678 Control: *n* = 664 *N* = 1,342	Edinburgh Postnatal Depression Scale (EPDS)	T1: Gestational weeks (gw) 21–25 T2: Gw 37 T3: 6 weeks after birth T4: 3 & 6 months after birth	Mamma Mia group displayed fewer depressive symptoms compared to the participants in the control group during follow-up.	No
**7**	Yang M et al. [29]	Online mindfulness intervention (Smartphone based)	China	Mindfulness (mobile based)	Mindfulness practices: Body screening Relaxation Meditation Consists of four sessions delivered via video recordings by trained nurses and shared on the WeChat platform in the form of text, pictures, and audio recordings.	RCT	Antenatal person with depression and anxiety: Intervention: *n* = 62 Control: *n* = 61 *N* = 123	Generalized Anxiety Disorder Scale (GAD-7) Patient Health Questionnaire (PHQ-9)	T1: At baseline T2: After intervention (8 weeks)	Participants in the intervention group showed greater declines in depressive and anxious symptoms compared with those in the control group.	No
**8**	Guo L et al. [30]	Mindful self-compassion intervention	China	6-week Internet-based Mindful Self-Compassion Program (web-based)	Tool to assess mindfulness and self-compassion: Mindfulness Attention Awareness Scale (MAAS) and Self-Compassion Scale (SCS)	Two-arm, open-label RCT	Antenatal person with depression: Intervention: *n* = 144 Control: *n* = 140 *N* = 284	Parenting Stress Index (PSI) EPDS State-Trait Anxiety Inventory–I and II Beck Depression Inventory II (BDI) Mindfulness Attention Awareness Scale (MAAS)	T0: At baseline (in the 2nd or 3rd trimester of pregnancy) T1: 3rd month postnatal T2: 1 year postnatal	Intervention group showed significant improvement in depressive and anxiety behaviors compared to control group.	No
**9**	Loughnan SA et al. [31]	Internet-delivered cognitive behavioral therapy (CBT) – MuMentum Pregnancy Program	Australia	iCBT (MUMentum) (web-based)	Intervention included: Psychoeducation Controlled breathing Progressive muscle relaxation Thought challenging Coping cards Structured problem-solving Activity planning and monitoring Graded exposure Assertive communication Relapse prevention Sleep hygiene Medication for anxiety and depression during pregnancy and breastfeeding Fight-or-flight response Pleasant activities Further skill examples Understanding intrusive thoughts and images Self-care plan The content was presented via illustrated stories displayed using slides.	RCT	Antenatal person with depression and anxiety: iCBT: *n* = 43 TAU: *n* = 44 *N* = 87	Generalized Anxiety Disorder 7 (GAD-7) Kessler Psychological Distress (K10) EPDS	T1: Pre-treatment T2: Post-treatment T3: 4 weeks follow-up	There was a significant reduction in anxiety on GAD-7 and psychological distress on the K10. iCBT was an acceptable treatment for antenatal anxiety and/or depression.	No
**10**	Krusche A et al. [32]	Online mindfulness	United Kingdom	Be Mindful Online (web-based)	Participants learned how to apply formal and informal meditation practices via videos and assignments that include body scan, mindful movement, breathing space, and mindful eating. Consists of 10 interactive sessions: **W0: Introduction** Course preparation and orientation Stress, anxiety, and depression assessment **W1: Stepping out of Automatic Pilot** Online session: Dealing with Barriers Assignments: Routine Activity, Mindful Eating, Body Scan Emails: Practicing at Home, Mindful Meal Anecdote **W2: Reconnecting with Body & Breath** Online session: The Physical Barometer Assignments: Mindful Movement, Event Awareness, Mindful Breathing Emails: Breathing Tips, Remember Your Intentions **W3: Working with Difficulties** Online session: On Negative Thoughts Assignments: Breathing Space, Stress Awareness, Sitting Meditation Emails: The Guest House Poem, 3-minute Breathing Space **W4: Mindfulness in Daily Life** Online session: Mindful Walking Assignments: Activity Awareness, Breathing Space and Action Step, Stress Strategies Emails: Preparing for Stress, Fear, and Fearless Quote **W5: Going Forward** Online session: Completion Certificate and Additional Resources	RCT	Healthy antenatal person: Intervention: *n* = 16 Control: *n* = 32 *N* = 48	Perceived Stress Scale (PSS) General Anxiety Disorder-7 (GAD-7) EPDS Tilburg Pregnancy Distress Scale (TPDS)	T0: At baseline T1: post-course T2: 8 weeks postnatal	There was a significant reduction in the intervention group in depression, pregnancy-related distress, and labor worry compared to the control group from T0 to T1.	No
**11**	Forsell E et al. [33]	Internet-delivered CBT	Sweden	iCBT (web-based)	**Module 1: Introduction** Psychoeducation about depression, antenatal depression, CBT, and the treatment platform. **Module 2: Being pregnant** Information about myths, facts, and the in-between concerning pregnancy-related physiological and cognitive changes, views, and stigma around antenatal depression. **Module 3 &4: Behavioral activation** Psychoeducation and rationale (e.g., how to conceptualize depression from a CBT perspective and why and how the suggested methods might work). Focus is on positively reinforced behaviors More behavioral activation, focusing on negatively reinforced behaviors, avoidant behaviors, and procrastination. **Modules 5 & 6: Cognitive restricting** Psychoeducation about negative automatic thoughts and acceptance Working with negative automatic thoughts Cognitive biases and traps, assumptions, and how to challenge them. Problem solving.	RCT	Antenatal person with major depression: iCBT: *n* = 22 TAU: *n* = 20 *N* = 42	EPDS Montgomery-Asberg Depression Rating Scale-Self Report (MADRS-S) Insomnia Severity Index (ISI) Work and Social Adjustment Scale (WSAS)	T0: At baseline T1: 3–6 weeks postnatal	Post-treatment depressive symptoms in the iCBT group were significantly lower compared to the TAU group.	No
**12**	Milgrom J et al. [34]	Internet cognitive behavioral therapy (iCBT)	Australia	MumMoodBooster (web-based)	The six sessions include: **Session 1:** Getting Started **Session 2:** Managing Your Mood **Session 3:** Increasing Pleasant Activities **Session 4:** Managing Negative Thoughts **Session 5:** Increasing Positive Thoughts **Session 6:** Planning for the Future The content for each session was presented using text, animations, video introductions, case vignettes, and audio and video tutorials. Consists of partner's website, coach's website, and administrative website	2 parallel-group RCT	Postnatal person with depression: iCBT treatment: *n* = 21 Treatment as usual: *n* = 22 *N* = 43	Beck Depression Inventory (BDI-II) Patient Health Questionnaire (PHQ-9) Depression Anxiety Stress Scale (DASS)	T1: At baseline T2: 12 weeks after enrollment	Depression symptoms of BDI-II in the intervention group decreased compared to the TAU group after 12 weeks.	Yes

## 4. Findings

Of the 12 studies, seven web-based interventions were implemented on a computer, whereas the other five utilized mobile applications for the implementation of their interventions. The studies reported a wide range of outcomes, including depression and anxiety symptoms. To assess depressive symptoms, most studies used the Edinburgh Postnatal Depression Scale (EPDS), followed by the Patient Health Questionnaire (PHQ) and the Beck Depression Inventory-II (BDI-II). To assess anxiety, the common measurement tools used were the Generalized Anxiety Disorder-7 (GAD-7) and the Depression, Anxiety, and Stress Scale (DASS). The less frequently used screening tools were the Montgomery-Asberg Depression Rating Scale-Self Report (MADRS-S), Perceived Stress Scale (PSS), Kessler Psychological Distress Scale (K10), State-Trait Anxiety Inventory–I and II, State-Trait Anxiety Inventory (STAI), Hamilton Depression Rating Scale (HDRS), and Inventory of Depression and Anxiety Symptoms (IDAS).

For the 12 RCTs, the assessment periods ranged from a few months up to two years. Interestingly, some studies with a larger sample size had relatively shorter study periods compared to those with a smaller sample size [24]–[29]. The duration of the interventions also varied, with most studies conducting assessments at three time points [24],[27],[30]–[32]. The remaining studies conducted assessments twice [26],[29],[33],[34], four times [28], and up to five times [23],[25] between the baseline and the end of the study period.

In terms of the sample population, three studies focused on healthy antenatal persons [26],[28],[32], and three focused on antenatal persons with depression [25],[30],[33]. Only two studies included antenatal persons with both depression and anxiety [29],[31]. Two studies focused on healthy postnatal persons [23],[24], and two focused on postnatal persons with depression [27],[34].

In terms of the intervention approach, there were no physical or face-to-face approaches since this review included only studies using digital or online interventions. Most studies used cognitive behavioral therapy (CBT) [23],[31],[33],[34] or mindfulness therapy [24],[29],[32] as interventions. Some researchers used a combination of CBT and mindfulness as an approach [25], while another applied a combination of mindfulness and self-compassion as the intervention [30]. Several studies failed to mention the exact therapeutic approach applied in the intervention. These studies labeled their approaches as psychoeducation, peer support intervention, and internet-based intervention [26]–[28].

The content of the CBT interventions included in the reviewed studies included breathing retraining, relaxation techniques, activity planning and monitoring, assertive communication, sleep hygiene, relapse prevention, fight-or-flight response, management of anxiety and depressive symptoms, self-care planning, and reducing negative and increasing positive thoughts [23],[31],[33],[34]. With a mindfulness intervention, mindfulness-based activities, such as movement, eating, breathing, listening, body screening, relaxation, meditation, sleeping, and mindfulness education, are emphasized [24],[29],[32]. In addition, some studies encompassed social support, such as peer support, partner support, trained peer volunteers, and phone coaching. However, of the 12 papers reviewed, only one included partner participation in the intervention.

In summary, we report beneficial outcomes in terms of improved depression and anxiety scores compared to the baseline assessment after the intervention [25],[26]–[29],[34]. Additionally, several studies reported significant differences in anxiety and depression levels before and after the intervention [24],[32]–[34]. Only two studies reported no difference in anxiety [26]. The beneficial outcome in this context refers to digital intervention studies that showed improvements in perinatal depression and anxiety. Most of the studies were based on the subjects' positive outcomes or feedback in terms of the reduction of their level of depression or anxiety at different time points.

## 5. Discussion

In this review, iCBT was found to be the most common intervention used for reducing perinatal depression and anxiety. Similarly, a previous systematic review identified CBT behavioral activation (BA), interpersonal psychotherapy (IPT), and mindfulness-based interventions (MBI) as the most common methods of treatment for perinatal depression [35]. iCBT was found to be effective in reducing stress, anxiety, and depressive symptoms in postnatal persons [36]. Nishi D et al. [37] indicated the effectiveness of iCBT in preventing antenatal depression and, therefore, postnatal depression after childbirth. iCBT also appears to be an effective intervention method during the perinatal period [38]. Stentzel U et al. [39] emphasized that iCBT, in both clinician-guided and self-guided formats, was successful in treating perinatal depression and anxiety. The benefits of iCBT, which surpassed those of face-to-face therapy, included fewer suicide attempts, less suicidal ideation, and less self-harm [40].

Correspondingly, Kumar V et al. [41] reported that both social functioning and patient knowledge could be improved via the use of iCBT. According to Li X et al. [42], both in-person groups (subjects and partners together) and individual person and partner (individually) formats were effective in improving perinatal depression in the short term. Thus, partner-inclusive CBT that focuses on couple relationships and social support from partners should be advocated and made feasible. Partners were taught to provide support and to prevent conflict or distress in postnatal subjects undertaking CBT sessions [42]. Chen C et al. [43] reported that both mindfulness therapy and CBT interventions significantly decreased depressive symptoms in the intervention group compared with those in the control group. However, it remains inconclusive whether iCBT is more successful than other therapies in addressing perinatal depression compared to anxiety. Hence, more studies are warranted to establish the most effective therapeutic interventions for different target populations.

Furthermore, we found that the majority of perinatal persons preferred a digital intervention because this helped them overcome certain barriers related to physically seeking help. Our results are consistent with those obtained by Neo HS et al. [44], who reported two major categories of help-seeking-related barriers, namely, structural and psychological barriers. While structural barriers refer to insufficient human resources (e.g., a lack of mental health professionals) and access difficulties (e.g., limited appointment slots), psychological barriers include a lack of motivation or self-perceived barriers to seeking treatment. Similarly, Martinengo L et al. [45] reported that traditional face-to-face CBT can be costly, time-consuming, and subject to the availability of trained providers, thus limiting its accessibility to a wide range of patients. As an alternative, iCBT programs have been designed as acceptable and effective alternatives to improve accessibility to therapy [46].

Apart from improving access from the patient's perspective, digital interventions enable healthcare practitioners to provide outreach for patients in need [47]. They allow a person to easily seek help without visiting a healthcare facility or physically meeting a healthcare practitioner. Thus, iCBT can offer significant user convenience by allowing more therapy choices and reducing transportation and wait times, not to mention better protection of patient privacy [46]. Rogers MA et al. [47] also reported that digital approaches would be more appealing to perinatal persons who feel less comfortable in public places or those reluctant to seek treatment due to social stigma. Undeniably, internet-delivered interventions offer greater flexibility, which is vital for people who require improved access to CBT during the demanding peri- and postnatal periods [48]. Moreover, during the Covid-19 pandemic, digital platforms were much preferred over face-to-face interaction due to the fear of infection [46]. Nevertheless, more studies are needed among perinatal persons with depression and anxiety to establish the relative effectiveness of digital over face-to-face interventions.

However, it is crucial to consider the non-significant difference in terms of time interaction for both depression and anxiety when comparing intervention and control groups. This review highlighted that depression and anxiety levels in the intervention groups were lower than those in the control or standard treatment groups. Most studies included in this review showed a variety of results between the group assessments from baseline to post-intervention. Moreover, almost all the reviewed studies reported a significant difference in depression and anxiety over time between the intervention and control groups. For instance, Suchan V et al. [49] highlighted that patients with postnatal depression showed symptomatic relief that was compatible with the treatment responses reported in the literature investigating face-to-face CBT. Based on these findings, it can be postulated that digital interventions lead to better outcomes for both antenatal and postnatal persons with depression and anxiety. That being said, researchers should focus on the effectiveness of digital interventions that encompass partner involvement, especially in terms of time interaction.

With regard to efficacy, online-based CBT for intervention groups was more effective compared to those undergoing treatment as usual or in control groups. If digital interventions can be integrated into existing treatment plans, the burden on HCPs can be greatly reduced. In a recent study, iCBT usage was associated with greater effectiveness in reducing postnatal depression compared to traditional treatment [50]. Zhao L et al. [51] also demonstrated that postnatal depression levels in their telehealth group were lower than those in the control group after the intervention, indicating that digital interventions can be more effective in reducing depression than the usual treatments. Notably, almost all the studies in the review involved both healthy perinatal persons as well as perinatal persons with depression and anxiety symptoms.

Overall, digital interventions conferred more benefits on subjects with depression and anxiety than on those who were healthy. A published study showed that when iCBT was provided under the guidance of a clinician, significant effect size reductions were observed in the symptoms of depression and anxiety among antenatal patients [52]. Although adherence to unguided iCBT has been reported to be lower in some studies, it is comparable to guided iCBT in reducing anxiety and depression in the general adult population [53]. In short, whether or not a digital intervention confers a better outcome on persons with perinatal depression and those with anxiety compared to their healthy counterparts remains inconclusive. Therefore, more research is required among healthy perinatal subjects to better delineate the effectiveness of digital interventions in alleviating depression and anxiety when compared with healthy perinatal persons.

In this review, a common observation in the included studies was the lack of elements of partner support or participation. Only one study involved partners in digital intervention. Levels of stress and depression in perinatal persons tend to increase if they receive low levels of support from their partner, subsequently compromising their quality of life [54]. As such, partner participation or involvement is crucial in reducing depression and anxiety among perinatal persons, thus necessitating more studies on how partner participation or support could alleviate perinatal depression and anxiety symptoms.

There are several strengths to this review. First, comparing healthy persons to those with clinical depression and anxiety in terms of the efficacy of the therapies is advantageous. Most studies involving clinical populations (e.g., perinatal subjects with depression and anxiety) produced more significant improvements compared to improvements in healthy perinatal persons, thus emphasizing the significant effects of the interventions in reducing depression and anxiety symptoms. In addition, this review provides the latest updates on the effectiveness of digital interventions in addressing perinatal depression and anxiety.

Nevertheless, this systematic review has several limitations. First, only studies published in English or Malay were included, so other relevant papers published in other languages might have been excluded. Reviews should be more inclusive by including studies published in other languages to generate a comprehensive view of the effectiveness with which digital interventions address perinatal depression and anxiety. Moreover, almost none of the studies focused on partner-inclusive intervention; thus, more studies should be conducted to gather more evidence regarding the effectiveness of partner involvement. In addition, a total of 55 articles could not be retrieved because full access was prevented due to a limited subscription. Some of these papers might contain additional information related to perinatal depression and anxiety. Finally, a poor internet connection or poor access to Wi-Fi or broadband when using online interventions was one limitation that should be considered in the context of this study. Therefore, authorities (i.e., developers of mental health apps) should consider the limitations of similar low-resource settings when designing mental health apps, ensuring that data can be saved and used offline. Thus, more studies, especially those related to partner-inclusive interventions, are warranted to generate better reference material for local settings. Furthermore, we did not include intimate partner violence among the study criteria, as its presence in any relationship may affect the mental well-being not only of the pregnant person but of the couple. This, in itself, may be a confounding factor that would affect the results of the study. Therefore, we focused only on partner support, investigating its potential for use in future interventions. Studies that were included in the reviews were mainly primary interventional studies published between 2016 and 2023 that were not limited to any specific countries. However, the studies from the United States were excluded because they were pilot studies, and no results were included in those papers.

In the study conducted by Stoll CRT et al., it was recommended to include two (or more) reviewers in the screening process to reduce or prevent any misapplication of the eligibility criteria as well as random errors by a single reviewer [55]. The present review included only two reviewers in the data extraction process. Future reviews should consider including more than two reviewers to prevent observational bias.

## 6. Conclusion

In summary, this study provided a clear review of the content and approaches related to existing interventions for depression and anxiety among perinatal subjects. Certain methods, such as iCBT and mindfulness, were commonly applied as interventions in the included studies. In contrast, the element of partner participation has not been fully integrated into existing interventions. These findings should be taken into consideration by the relevant stakeholders when designing and implementing the necessary steps to mitigate perinatal depression and anxiety.

## Use of AI tools declaration

The authors declare they have not used artificial intelligence (AI) tools in the creation of this article.
